# Medical Opinion and Movement

**Published:** 1911-02-25

**Authors:** 


### Medical Opinion and Movement.

### Salvarsan for Inherited Syphilis.

Amid the flood of papers dealing with the effects of "606," or salvarsan as it is now officially termed, are several dealing with its influence on inherited syphilis in the newly born. Ehrlich himself has advised that such infants should not be treated directly with his remedy, but through the mother or through a (syphilitic) wet-nurse. He says that after direct treatment children may improve for a day or two, but then die as a result of the liberation of toxins set free by destruction of myriads of spirochætes, whereas when salvarsan is given to the mother she produces antitoxins, which reach the child through the milk. After the temporary improvement which then ensues it is possible to give the treatment directly to the child without danger: the dose for infants is .01 gram per kilogram of body weight. Duhot reports a case which bears out this contention. He treated a mother for obstinate syphilis three weeks after the birth of her child with two doses of salvarsan, .95 gram in all. Not only did the mother improve rapidly, but the child, which was not thriving and exhibited typical lesions, at once began to take well and to put on weight rapidly; the symptoms of syphilis in it disappeared also. Rosenthal, however, reports a similar sort of case in which 606 given to the mother had no effect on the child, which, on the contrary, got worse. The familiar mercury treatment cured it speedily in a most satisfactory way. Kalb, again, believes in salvarsan given direct to babies: he reports five cases, with one death. In the four survivors the results, as recorded, are satisfactory; and Kalb himself seems not to admit that the treatment was responsible for the single fatality.

### The Nauheim Baths.

Writing in the *Medical Record*, Dr. Cornwall, of Brooklyn, has some forcible remarks on the subject of formal exercises, massage, and baths in the treatment of cardiac disease, with especial reference to what is commonly known as the Nauheim system. He has for many years held and expressed the opinion that this remedy is greatly over-rated; that its usefulness is limited, at the best, to a very small class of cases; and that the inconveniences, and even danger, which attend its use, except in the most carefully selected cases, are sufficient to warrant a disregard of the large claims made for it by its promoters, notwithstanding the extensive approbation with which those claims have been received by the medical profession. The treatment, he says, is not generally used by doctors, though the Nauheim baths figure in text-books as a standard remedy whose value is unquestioned. He prophesies that the time is not far off when text-books will give them less conspicuous mention, and perhaps relegate them to the historical sections. It is interesting to compare with this outspoken declaration the views of Dr. Jas. Mackenzie, as expressed in his recent work on "Diseases of the Heart." "I feel it would be misleading if I passed in silence a method of treatment that has obtained a world-wide reputation which I consider out of proportion to its merits. . . . . I am impressed with the injury done to individual patients through the unmerited reputation of the Nauheim baths among the medical profession. In the patients entering the baths I could discover no improvement from the single immersion. I may frankly confess that I saw no patient get benefit at Nauheim that would not have done equally well elsewhere."

### The Respiratory Complications of Ether.

One of the objections raised against ether as an anæsthetic has always been that it is more liable than chloroform to give rise to pulmonary complications. Since the introduction of the open method of administration it has been asserted that this danger is more or less imaginary, and that when given in this way there is not the same incidence of these formidable complications as under closed methods. In the *Boston Medical and Surgical Journal* Dr. Graves records two thousand consecutive administrations of open ether. Out of the whole number there were twenty-two cases of subsequent pulmonary trouble: eight of broncho-pneumonia, ten of bronchitis, four of pleurisy. It is further noteworthy that twenty of these twenty-two were laparotomy cases, though almost half of the operations were of other kinds. So that the incidence of pulmonary complications is nine times as high in the laparotomy series as in the other. He concludes that post-operative lung complications occur more frequently and are more fatal in cases already septic or desperately sick, and in the aged and feeble. Shock, duration of anæsthesia, method of administration, embolism, wound sepsis, and other like factors seem to have no causal relation to post-operative pneumonia. The latter and bronchitis are rare during the summer and frequent during the winter months. Pre-existing foci of infection in the lungs are almost invariably lighted up or aggravated by ether anæsthesia, but could be avoided by more careful examination of the respiratory tract after operations. The extent to which the morphia, atropine, or other drugs given hypodermically before the open ether method affect the problems of lung trouble might with advantage be considered. On the one hand, morphia must tend, even if but slightly, to respiratory hypostasis, and, on the other, it reduces so greatly the quantity of ether inhaled that it must tend to much less irritation of the respiratory passages.

### Cancer and Infectious Diseases.

Attention has often been drawn to the antagonism which appears to exist between cancer and certain infectious diseases. Rokitansky considered there was a marked antagonism between cancer and tuberculosis, and more recently Löffler, observing the rarity of cancer affections in certain tropical regions, such as the island of Borneo, in which, on the other hand, malaria is so prevalent, suggests that malarial disease in some way excludes the occurrence of cancer. In the *Medizinische Klinik*, Dr. R. Schmidt seeks to extend this field of antagonism more widely. By careful inquiry into the previous history of a number of patients afflicted with cancer he finds that a large proportion of them had always had good health and had never suffered from any infectious disease. His inquiry extended over 241 cases of cancer of the digestive tract, including 198 cases of cancer of the stomach. Of these 180 had never suffered from any infectious disease during their childhood, and 99 of them had no infectious malady in the course of their subsequent life. The remaining 81 had only suffered from some adult infection. Of the sixty-one patients who had suffered from some infectious disease in childhood forty-nine had had only one infection of this kind, and thirty-one of these had been quite free from any subsequent adult infection, and only three patients had suffered from several infections. The author suggests the term "index of infection" to denote the number of infections that a patient has been subject to, and it is evident from these figures that the index of infection of these cancer patients was excessively low, especially in regard to the infectious diseases of childhood. Dr. Schmidt is disposed to the idea that those for whom this index of infection is low or is equal to zero are especially predisposed to cancer. Two theories are suggested to explain this antagonism between the infectious fevers and cancer. Either the predisposition to cancer constitutes a sort of diathesis which involves more or less immunity to the infectious diseases, or the infectious processes exercise some modifying influence on the organism, rendering it less liable to cancer affections; that is to say, the infectious diseases are in some measure prophylactic against cancer. According to this theory the efforts of modern hygiene, in so far as they tend to stamp out the infectious disease, will at the same time lead to an increase in the occurrence of cancer—an unpleasant conclusion which one would fain hope is erroneous.

### Autotherapy for Ascites.

An account has already appeared in these columns of the method of treatment of cases of chronic pleuritic and ascitic effusions by subcutaneous or intravenous injections of the fluid previously drawn off under aseptic precautions. At a meeting of the *Société Médicale des Hôpitaux*, Dr. Sicard reported two cases of ascites from atrophic cirrhosis of the liver which he has treated by intravenous injections of large quantities of the fluid into the patients. Every ten or twelve days 300 to 500 grammes of the fluid were drawn off and immediately injected into a vein in the arm. No improvement was observed till after the fifth or sixth injection. One of the patients treated in this way may be considered cured, the other is still under treatment. Both patients were treated previously by simple puncture, diuretics, dechlorinated diet, etc., without any improvement. The author points out that the only necessary precaution to take before having recourse to this method of autotherapy is to make sure that the ascitic fluid is sterile by means of inoculations into guinea-pigs.

### New Signs of Pleuritic Effusion.

In the French current medical literature attention has been drawn to two new signs which may prove of clinical value in the diagnosis of pleuritic effusions. The first one has been demonstrated by Dr. Felix Ramond and an account of it appears in *Le Progrès Médical*. He calls it "the sign of the spinal muscles"; it consists in the fact that in a case of pleurisy the muscular mass constituted by the long dorsal and iliocostal of the affected side is in a state of permanent reflex contraction. On inspection the muscular mass, especially in the lumbar region, appears more prominent and swollen on the affected side. On superficial palpation, and more especially on deep palpation, the muscles feel hard and resistent, comparable with a slightly stretched cord of indiarubber and quite different from the softer feeling of the muscles on the sound side. If the upper attachments of the long dorsal at the sixth and seventh dorsal vertebræ be percussed, and at the same time the hand be applied to the muscular mass in the lumbar region, the muscle is felt to vibrate and the reflex contraction may be so strong as to be visible to the naked eye. This last sign may, however, be dependent upon an exaggerated muscular reflectivity, apart from any pleuritic effusion. The difference between the two sides is best brought out by making the patient bend first to one side and then to the other. Under normal conditions the muscles on the flexed side completely relax, but in the presence of pleurisy they do not lose their tonicity in flexion of the same side, and in flexion of the opposite side they are exaggeratedly firm and contracted. This abnormal condition of the muscles persists often for some weeks after other signs of pleurisy have disappeared so that this muscle sign may serve to afford a retrospective diagnosis.

The second new sign is described by Dr. P. Mauriac. The patient should be seated or standing and the physician places his hand on the lower part of the chest, so that the thenar eminence rests on the hypochondrium and the pulp of the middle or index finger in the sub-xiphoid fossa. In this position the medius or index finger percusses lightly, the percussion being performed with the pulp and not with the tip of the finger. In this way a hard clapping sound should be obtained. At the same time the stethoscope is applied to the back of the chest, first to one side and then to the other. On the side where there is no effusion a heavy, distant, muffled sound is heard, but on the side of the effusion there is a hard clapping, almost metallic bruit. Examination for this sign requires practice, especially to obtain the proper percussion, which necessitates a certain elasticity of the finger. The author points out that the sign has the advantage of not requiring any instrument or assistance, and as the percussion point is the same for both lungs comparison is much simpler and much more exact.

### Double Appendicitis.

In the *Journal of the American Medical Association* is recorded by Dr. Young a case in which two appendices were found at operation, both suppurating. One measured 3½ inches in length, the other 3 inches. One of them had ruptured, the other not. Each appendix had its own meso-appendix, and there was 1¼ inch between the two bases. Both appendices were removed, and the abscess was drained. Continuous proctoclysis was also instituted, and the Fowler position adopted. Recovery was slow, and the patient's condition was serious for the first five days. Eventually a complete return to health took place.

### Splenic Anæmia.

After a lot of experimental work on animals, conjoined with clinical studies, Dr. Warthin concludes that the symptom-complex of splenic anæmia and Banti's disease, as formulated by Osler, is in certain cases, if not in all, the result of obstruction of the portal and splenic circulation. It is the splenic obstruction which counts for most; and it is old thrombo-phlebitis which is most often at the bottom of the subsequent trouble. Other causes will, however, produce the same effect: artificial compression of the portal and splenic veins, torsion of the latter, dislocation of the spleen, etc. The essentials of all three lesions—splenic anæmia, Banti's disease, and old porto-splenic thrombosis—are the same: large spleen, small liver, hæmatemesis, hæmorrhages from mouth and rectum, ascites relieved by hæmorrhage, intermittent course due to progressive thrombosis with canalisation, anæmia, and leucopenia. More rare are icterus and very acute or very chronic course. The whole pathological picture points to an infective thrombo-phlebitis of the portal and splenic veins as the essential feature of all the cases, no matter how they are reported. Syphilis is probably the most frequent cause; pyogenic infections explain others; and, in the author's opinion, the symptom-complex may be a sequel or complication of a number of the infections. The original paper is to be found in *International Clinics*.

### Congenital Family Cholæmia.

A case of this peculiar condition is fully reported in the *Edinburgh Medical Journal* by Drs. Mackintosh, Falconer, and Anderson. The patient is a boy of seven years, referred for examination, not because of any symptoms of illness, but picked out by a school medical officer for investigation. He is the third child of healthy parents, and has been jaundiced since within a few weeks of birth. The eldest child died at six months, having been deeply jaundiced during the whole of that time; the second child is normal. The patient's icterus varies in degree from time to time; when it increases he is easily tired and drowsy, but in the usual way he has no symptoms at all. He has passed successfully through an attack of measles. He has never had any epistaxis or other hæmorrhages. The spleen is much enlarged and extends an inch below the costal margin; there is no other abnormality to be discovered, save in the examination of the blood, which shows only 2,800,000 red cells and 35 per cent. of hæmoglobin; there is also a definite tendency to microcytosis and polychromatophilic degeneration. The leucocytes are about normal; the blood serum contains bile pigment. The urine is not abnormal and never contains bile pigments; the stools are well coloured, and when the jaundice is most marked they are dark green. The liver is apparently normal. This syndrome is one of which a great many cases have now been published since Nimkowski first described it in 1900. The cases vary in details, but this is a fairly typical case in all essential respects. The condition affects, and can be transmitted by, both sexes; it is compatible with long life, for cases have been reported at seventy-four, seventy-four, and eighty-one. In cases which have come to autopsy the chief findings have been perisplenitis, with increase of the spleen pulp due to engorgement, with red cells. Gall stones have been reported five times. Most writers agree to ascribe the condition to hæmolysis and diminished resistance of the red cells.

### Auscultation of Joints.

In a paper read before the Royal Society of Medicine, and printed in the *Lancet*, Dr. Garrod describes the diagnostic uses of auscultation when dealing with diseases of joints. An ordinary stethoscope is used; after a little practice it is possible to dispense with an extra small chest piece, a rubber ring, or other aid to coaptation. The knee is the joint which best lends itself to auscultation; at either side of the patella it is easy to apply the stethoscope, and to hear abnormal sounds when the joint is flexed and extended. Most of the other large joints can be similarly studied, and even some of the small joints also. A normal joint in a young subject is silent, save for the loud clicks which some people can produce at will; these are vacuum sounds. In those past middle age there may be abnormal sounds when the patient is quite unconscious of any articular disability; probably these are premonitory signs of osteoarthritis. A distended joint is noiseless, but crackles appear as the fluid is absorbed and the synovial surfaces are once more in contact; these noises resemble those heard with the stethoscope over the site of surgical emphysema. Tubercular joints are usually silent, and so are those in the acute forms of rheumatoid arthritis: in both diseases the main stress of the inflammation is in the periarticular tissues. Scrunching sounds are typical of osteo-arthritic changes, erosion, and such like. A rubbing, friction-like sound is heard in late stages of rheumatoid arthritis; and just as characteristic is another sound which can be heard over inflamed tendon sheaths. The author lays down no definite rules, but suggests that further study may tend to establish the method as one valuable in the differentiation of articular diseases.

